# Umbilical cord blood-based gene signatures related to prenatal major depressive disorder: Retraction

**DOI:** 10.1097/MD.0000000000019445

**Published:** 2020-02-21

The article, “Umbilical cord blood-based gene signatures related to prenatal major depressive disorder”^[1]^ which appears in Volume 98, Issue 28 of *Medicine*, is being retracted over concerns regarding unsuitable use of the GEO differential expression tool and the resulting unsupported conclusion that “This bioinformatic analysis demonstrated immune-mediated mechanisms might play a fatal role in abnormalities in fetal gene expression profiles caused by prenatal MDD.”
